# Diet, lipids, and antitumor immunity

**DOI:** 10.1038/s41423-021-00781-x

**Published:** 2022-01-05

**Authors:** Hannah Prendeville, Lydia Lynch

**Affiliations:** 1 Trinity Biomedical Sciences Institute, Trinity College Dublin, Dublin, Ireland; 2 Brigham and Women’s Hospital, Harvard Medical School, Boston, MA USA

**Keywords:** Lipids, cancer, obesity, β-oxidation, anti-tumour immunity, Tumour immunology, Cancer metabolism

## Abstract

Tumour growth and dissemination is largely dependent on nutrient availability. It has recently emerged that the tumour microenvironment is rich in a diverse array of lipids that increase in abundance with tumour progression and play a role in promoting tumour growth and metastasis. Here, we describe the pro-tumorigenic roles of lipid uptake, metabolism and synthesis and detail the therapeutic potential of targeting lipid metabolism in cancer. Additionally, we highlight new insights into the distinct immunosuppressive effects of lipids in the tumour microenvironment. Lipids threaten an anti-tumour environment whereby metabolic adaptation to lipid metabolism is linked to immune dysfunction. Finally, we describe the differential effects of commondietary lipids on cancer growth which may uncover a role for specific dietary regimens in association with traditional cancer therapies. Understanding the relationship between dietary lipids, tumour, and immune cells is important in the context of obesity which may reveal a possibility to harness the diet in the treatment of cancers.

## Introduction

The rate of cancer growth and effectiveness of antitumor immune responses are determined by nutrient availability in the tumor microenvironment (TME). There is vast literature on the metabolic environment of tumors; on one hand, studies describe tumors as having low metabolic activity with low levels of glucose and glutamine [1]. On the other hand, other studies describe high glucose levels in tumors [2]. Recently, however, several studies have begun to shed light on the lipid composition of tumor interstitial fluids (TIFs). Lipid abundance is now recognized as a feature of many tumors [3, 4] and lipids have emerged as a potential enemy of an antitumor environment. This review will describe the main mechanisms by which lipids support tumor growth and metastasis while also fostering an immunosuppressive environment.

Lipids are a heterogeneous pool of water insoluble metabolites that mainly comprise cholesterol and fatty acids (FAs). Cholesterol is a cyclic sterol that can be obtained through dietary intake or can be synthesised via the mevalonate pathway. Endogenous cholesterol production primarily occurs in the liver and intestine, although all mammalian cells are capable of cholesterol synthesis. FAs are derived from dietary intake or synthesised de novo in the liver in response to insulin signaling after feeding [5]. FAs represent a major source of energy for cells but are also key components of complex structural lipids, including phospholipids and sphingolipids, which together with cholesterol comprise the major components of cell membranes [6]. In times of low energy demand, adipose tissues store excess FAs in the form of triacylglycerols (TAGs), which, along with cholesterol esters, are the main components of intracellular lipid droplets (LDs) [6]. LDs are dynamic organelles that promote lipid homeostasis by sequestering excess lipids to prevent lipotoxicity. TAGs in LDs can be mobilised through the action of lipases, releasing FAs that can be used to construct PLs for membrane formation or as substrates for β-oxidation [7]. An optimal supply of FAs is therefore essential for highly proliferative cells, such as cancer cells, to provide energy and building blocks to support their rapid growth.

Here, we will focus on the role of lipid metabolism in tumor and immune cells, which is also relevant in the context of obesity, in which there is an increased risk of many types of cancers and which is now a major issue worldwide. We will discuss the role that dietary fat plays in cancer initiation and progression in the context of obesity and propose the question of whether modifying dietary intake of fat may influence the efficacy of cancer therapy.

## Lipids and cancer

Given the dynamic roles of lipids in membrane production and energy generation, it is not surprising that lipid metabolism plays a significant role in cancer. To date, most evidence suggests that lipids promote the survival and spread of cancer [6]. Lipid abundance is now an established hallmark of many tumors, including melanoma [3], pancreatic cancer [8], and others. In murine models, the tumor interstitial fluid (TIF) of B16 melanoma tumors contains many FA species, which increase in abundance with tumor progression [3]. This lipid phenotype is common across other models of melanoma, including patient-derived melanoma xenografts and human melanoma metastases [3]. Similarly, in mouse models of pancreatic cancer, as pancreatic tumors develop, lipid droplets accumulate long-chain fatty acids (LCFAs), which promote a protumorigenic environment [8]. In addition, the TIF of B16 tumors also contains many  acylcarnitines, ceramides, phospholipids and esterified cholesterol, highlighting the diverse nature of lipids in the TME [4]. Here, we will describe how cancer cells acquire lipids and detail the recent findings on the consequences of rewiring metabolism toward lipogenesis and β-oxidation to support key oncogenic functions.

### Exogenous lipid uptake

Understanding how lipids accumulate in tumors may provide therapeutic opportunities to inhibit lipid uptake or metabolism. Cancer cells express a myriad of receptors and binding proteins to facilitate lipid uptake and trafficking, including CD36, low-density lipoprotein receptor (LDLR) and fatty acid binding proteins (FABPs). CD36 is a cell surface scavenger receptor involved in the recognition and delivery of a host of lipids to cells, including long-chain fatty acids (LCFAs) and oxidized low-density lipoprotein (OxLDL) [9]. Lipid uptake via CD36 and the induction of β-oxidation boosts the metastatic potential of human oral carcinomas, which is inhibited by anti-CD36 antibody therapy in orthotopic mouse models of human oral cancer. CD36 expression correlates with poor disease-free survival in patients with squamous cell lung carcinoma and bladder and breast cancers, possibly because the predisposition to accumulate and metabolise lipids initiates metastasis [10]. These studies stress the importance of lipid uptake in mediating tumor dissemination and raise the possibility that targeting CD36 to impair fatty acid uptake and metabolism may offer therapeutic benefit for many cancers.

LDLR is a cell surface receptor that binds circulating LDL to internalize FAs and cholesterol esters [11]. LDLR^hi^ human breast cancers have lower overall and recurrence-free survival, and LDLR^hi^ mouse models of breast cancer show resistance to chemotherapy and hormone therapy [12], possibly due to increased intratumoral cholesterol content. Indeed, hypercholesterolemia is a risk factor for estrogen receptor-positive (ER^+^) breast cancers [13], and intratumoral cholesterol ester accumulation is associated with increased breast cancer cell proliferation as assessed by Ki-67 staining [14]. High-grade human breast cancers exhibit high expression of cytochrome P450 27A1 oxidase (CYP27A1), which generates oxysterol 27-hydroxycholesterol (27-HC) from cholesterol [15]. 27-HC is an estrogen mimetic that increases the growth and metastatic potential of ER^+^ breast cancers. Pharmacological inhibition of CYP27A1 prevents 27-HC accumulation and attenuates breast cancer growth [15]. Blocking LDLR-mediated cholesterol uptake may also decrease 27-HC levels, which may be a useful strategy to prevent or treat breast cancer.

On the other hand, FABPs are intracellular lipid chaperone proteins that coordinate lipid trafficking and lipolysis [16] and have been implicated in human ovarian cancer. It appears that some cancers have learned to exploit their anatomical location to sequester and metabolise lipids from nearby fat depots. Human ovarian cancer cells preferentially metastasise and home to the omentum, where they express high levels of FABP4 to assist fatty acid transfer from local adipocytes, providing a proliferative advantage [17]. Inhibition of FABP4 prevented adipocyte-mediated invasion and lipid accumulation in human ovarian cancer cells in vitro, while FABP4 deficiency resulted in fewer metastatic nodules in the ovarian bursa in a mouse model of ovarian cancer [17]. That study established a metastasis-promoting role for lipid accumulation, FABP4 expression and lipid metabolism in ovarian cancer.

### De novo fatty acid synthesis

**Fig. 1:**
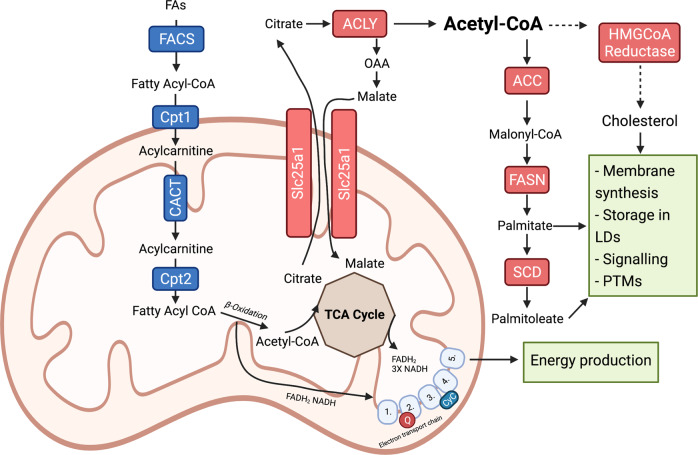
Overview of lipid metabolism. Lipid metabolism consists of two distinct arms, namely, fatty acid oxidation (β-oxidation) and lipid synthesis. The peroxisome proliferator-activated receptor (PPAR) family of transcription factors regulates the expression of genes involved in β-oxidation, as depicted in blue. However, the sterol regulatory element binding protein (SREBP) family of transcription factors controls the expression of enzymes and transport proteins required for fatty acid and cholesterol synthesis, as shown in red. Briefly, cytosolic fatty acids (FAs) are activated by fatty acyl-CoA synthetase (FACS), which introduces a CoA adduct, yielding fatty acyl-CoA. Long-chain fatty acyls enter the mitochondria for oxidation via the carnitine shuttle system. Carnitine palmitoyl-transferase 1 (Cpt1), located on the outer mitochondrial membrane, exchanges the CoA adduct for a carnitine molecule, forming acylcarnitine. Acylcarnitines are transported into the mitochondrial matrix through carnitine acyl-carnitine translocase (CACT), where they are reconverted back to fatty acyl-CoA by the action of Cpt2. Once inside the matrix, fatty acyl-CoA undergoes a series of dehydration, hydration and thiolysis reactions (β-oxidation), which generate NADH and FADH_2_ reducing equivalents and acetyl-CoA. NADH and FADH_2_ donate electrons to complexes 1 and 2 of the electron transport chain and acetyl-CoA enters the tricarboxylic acid (TCA) cycle. In the TCA cycle, acetyl-CoA is converted into citrate, which may be exported to the cytosol through Slc25a1. The enzyme ATP citrate lyase (ACLY) catalyses the conversion of cytosolic citrate to acetyl-CoA, which is an important step in endogenous lipid synthesis. Cytosolic acetyl-CoA is converted to malonyl-CoA through the action of acetyl-CoA carboxylase (ACC), which together form the major substrates for fatty acid synthesis by the multienzyme complex fatty acid synthase (FASN). FASN catalyzes the synthesis of palmitic acid, a 16 carbon saturated fatty acid, which may be converted into palmitoleic acid, a 16-carbon unsaturated fatty acid through the action of stearoyl-CoA desaturase (SCD). In addition, cytosolic acetyl-CoA may enter the mevalonate pathway, resulting in the production of cholesterol and other steroids. Together, fatty acids and cholesterol play an important role in membrane synthesis and signaling and can also be stored in lipid droplets to be later used as a fuel source

In addition to the uptake of exogenous lipids, many cancer cells are equipped to synthesise lipids endogenously (Fig. 1). Cytosolic acetyl-CoA is the major substrate for the synthesis of fatty acids, primarily those from glucose-derived carbons [18]. Glucose-derived pyruvate is metabolised in the tricarboxylic acid cycle (TCA cycle) to citrate, which is shuttled from the mitochondria to the cytosol via the mitochondrial citrate transporter (Slc25a1) [19]. Here, citrate is cleaved by ATP citrate lyase (ACLY), yielding oxaloacetate and acetyl-CoA [20]. Human breast carcinoma tissue and multiple mouse and human breast cancer cell lines have high expression of Slc25a1, which is enhanced by the activation of oncogenic signaling pathways, including the p53 pathway [21]. Slc25a1 overexpression likely favors a shift toward fatty acid synthesis and oxidative phosphorylation (OXPHOS) to decrease the reliance on glycolysis when extracellular glucose is limited [22]. It is also possible that in times of glucose abundance, glucose is directed toward pyruvate and citrate synthesis to make acetyl-CoA via Slc25a1, and thus, lipid metabolism could be favored in the tumor regardless of glucose levels.

While the mitochondrial citrate transporter is imperative to initiate fatty acid synthesis, carboxylation of cytosolic acetyl-CoA to malonyl-CoA by acetyl-CoA carboxylase (ACAC) is the rate-limiting step in this process (Fig. 1). ACAC expression is high in both advanced human breast carcinomas and preneoplastic lesions, highlighting the importance of fatty acid synthesis in multiple stages of cancer development [23, 24]. The final step of fatty acid synthesis, that is, the condensation of acetyl-CoA and malonyl-CoA to palmitic acid (C16:0), is catalysed by the major biosynthetic multienzyme fatty acid synthase (FASN). FASN expression is high in the liver, which is the main site for de novo lipid synthesis [20]. FASN is also highly expressed in stem and progenitor cells, which are highly proliferative and require lipids for membrane formation and energy production via β-oxidation [25]. Consequently, high expression of FASN is a feature of many cancers, including colon [26], prostate [27], and breast [28] cancers, and is associated with poor prognosis and disease-free survival. Similar to the endogenous biosynthesis of FAs in the liver, neoplastic lipogenesis is controlled by the sterol regulatory binding protein (SREBP) family of transcription factors. SREBPs are master regulators of lipogenesis and control the transcription of FASN in response to multiple environmental signals, including growth factor receptor activation of PI3K and MAPK pathways—pathways that are commonly hyperactivated in many cancers, including breast, endometrial and ovarian cancers [29]. In addition to supplying lipids for energy and membrane formation, another potential mechanism of FASN oncogenicity may involve palmitoylation—a post-translational modification of proteins involving the covalent attachment of palmitate to cysteine, serine, and threonine residues [30]. Indeed, FASN overexpression results in palmitoylation of Wnt1 and consequent activation of Wnt-β-catenin signaling, which regulates key cellular functions, including proliferation and differentiation, establishing palmitoylation as a potential oncogenic consequence of FASN overexpression [31]. Given these roles of endogenous fatty acid synthesis in cancer progression, FASN is an attractive target for cancer therapy. Cerulenin and C75 are potent small molecule inhibitors of FASN that have been shown to delay disease progression in models of ovarian and breast cancers [29]. Despite these promising therapeutic effects, targeting FASN alters multiple layers of lipid metabolism and homeostasis, which may result in unwanted side effects such as weight loss (possibly due to the cross activation of lipolysis and β-oxidation). Other, milder approaches such as dietary interventions may not have the same therapeutic power against cancer. For example, catechin is a natural flavonoid and antioxidant that can inhibit FASN without stimulating β-oxidation or inducing weight loss in mice [32]. Interestingly, catechin is a component of green tea, and as dietary manipulation is becoming an attractive strategy to enhance traditional cancer therapies, further research is warranted to fully elucidate the potential therapeutic effects of catechin as a FASN inhibitor.

### PPAR signaling and β-oxidation in tumor initiation, maintenance and dissemination

Glucose uptake and dysregulated anabolic metabolism in tumor cells have been a major focus for cancer researchers for decades. Tumor cells heavily rely on aerobic glycolysis (i.e., the Warburg effect) to facilitate biomass production and support their rapid proliferation [33]. Indeed, heightened glucose consumption forms the basis of ^18^F-deoxyglucose positron emission tomography (FDG-PET), a strategy used to detect and monitor tumor growth. However, the role of lipids and β-oxidation has recently gained traction in cancer biology. As cells proliferate and tumor volume expands, the metabolic landscape changes, resulting in vast areas devoid of nutrients, particularly glucose and glutamine [34], with maintained heightened levels of lipids [3]. To meet high bioenergetic demands, maintain ATP levels, and, importantly, proliferate, many tumor cells increase the expression of transcription factors and enzymes involved in β-oxidation. β-Oxidation is transcriptionally regulated by the peroxisome proliferator activated receptor (PPAR) family of transcription factors that sense fatty acid signals derived from dietary lipids and fatty acid metabolites [35]. In addition to cancer cells, many non-malignant cell types benefit from high levels of β-oxidation to maintain their function. Indeed, lipid oxidation is critical in the heart and liver. In stem cells, PPARδ signaling instructs the asymmetric division of hematopoietic stem cells to ensure the production of committed progenitors while replenishing the stem cell compartment. Inhibition of β-oxidation in vitro or in vivo ablation of PPARδ causes stem cell exhaustion and impaired maintenance of the stem cell pool, which may be involved in leukemogenesis [36]. Cancer stem cells (CSCs) are a subpopulation of tumor-resident cells capable of self-renewal and differentiation and have been implicated in tumorigenesis and metastasis initiation [37]. Human CSCs derived from colorectal adenocarcinomas adopt a β-oxidation gene signature and produce high levels of reactive oxygen species (ROS), likely due to aberrant lipid metabolism in these cells. This metabolic signature is responsible for promoting the metastatic potential of cancer stem cells by activating the epithelial-mesenchymal transition pathway, which potentiates cancer survival and metastasis [38]. Moreover, β-oxidation is essential to maintain the survival of human OCI-ALM3 and MOLM-13 leukemia cells, and treatment with etomoxir to inhibit β-oxidation in vitro sensitizes cells to apoptosis [39]. Together, these data demonstrate the importance of PPAR signaling and β-oxidation in supporting stem cell survival, maintenance, and function, establishing the FAO pathway as a potential therapeutic target for the treatment of hematological malignancies and other cancers.

An upregulation of PPAR signaling has recently been linked to the diet. Both cancer cells and cytotoxic lymphocytes, especially NK cells, can upregulate PPAR transcription factors in the settings of obesity and cancer [40]. PPAR signaling bestows adenoma-initiating properties upon intestinal progenitor cells, which is enhanced by high-fat diet (HFD) feeding. In mice, Lgr5^+^ intestinal stem cells proliferate and remodel the intestine in response to cues from the diet. Beyaz et al. found that in response to an HFD or following exposure to fatty acids, Lgr5^+^ cells have enhanced proliferation and stemness. This result was linked to the activation of PPARδ signaling within Lgr5^+^ intestinal progenitor cells, which resulted in aberrant proliferation and spontaneous formation of adenomas [41]. Loss of PPARδ or blockade of β-oxidation through inhibition of Cpt1a [42] blocks the proliferative effects of fatty acids on intestinal stem cells in vivo. These results highlight the role of lipid metabolism in initiating colon cancer in these models. Enhanced lipid metabolism has also been linked to the initiation of metastasis in other cancers. In human oral carcinomas, a tumor cell population that initiates metastasis is characterized by increased expression of CD36 and lipid metabolism genes [10]. PPAR transcription factors can induce the expression of CD36, further promoting lipid uptake to sustain β-oxidation. Indeed, lipid uptake though CD36 and induction of β-oxidation are critical for the survival and spread of squamous cell carcinomas [10]. Similar to the findings mentioned before, HFD feeding or treatment with palmitic acid enhanced the metastatic potential of the CD36^+^ metastatic cluster. While PPARs were not studied in this report, it is possible that, in addition to CD36, the full PPAR transcriptional program was activated in metastasis-initiating cells. Targeting PPARs could impair both cancer survival and spread by limiting the ability to acquire and metabolize lipids, inducing a metabolically vulnerable state.

In addition to aberrant PPAR signaling, many enzymes involved in β-oxidation are also dysregulated in cancer and contribute to high rates of proliferation, survival, and metastatic spread. The mitochondrial carnitine shuttle, composed of carnitine palmitoyl-transferase 1 (Cpt1), carnitine acylcarnitine translocase (CACT) and Cpt2, controls the rate of β-oxidation of LCFAs (Fig. 1). Cpt1 overexpression is a feature of many cancers, including human lung cancer, and offers a survival advantage by supporting ATP production through β-oxidation and resistance to glucose deprivation [43]. β-Oxidation is an aerobic process; thus, tumor cells relying on lipid metabolism require vascular network growth and optimal endothelial cell proliferation to support this process. β-Oxidation supports de novo nucleotide synthesis for DNA replication and proliferation of human endothelial cells, and blocking Cpt1a depletes nucleotide precursors in endothelial cells, inhibiting angiogenesis [44]. Therefore, other cells in the TME harness lipids from the TIF to fuel tumor-promoting processes. This is also true for tumor-infiltrating immune cells, which will be discussed in a later section.

### Cancer cells escape lipotoxicity

Cancer cells acquire,  synthesise, and metabolise lipids to support key oncogenic functions, including tumor initiation, proliferation, and metastasis. However, excess FAs and heightened β-oxidation can generate reactive oxygen species (ROS), which can trigger lipid peroxidation, cellular damage, ER stress and apoptosis [45]. Cancer cells have adopted many strategies to prevent lipotoxicity-induced cellular damage, which will be described in this section. At the same time, the tumor-infiltrating immune cells that are also exposed to excess lipids can undergo lipid peroxidation, which impairs their function.

Lipid peroxidation describes the oxidative degradation of lipids by free radicals. Polyunsaturated fatty acids (PUFAs) are prime targets for lipid peroxidation reactions due to the high proportion of carbon-carbon double bonds that house reactive hydrogen atoms [46]. The accumulation of lipid-based ROS triggers ferroptosis, a form of programmed cell death. Antioxidant molecules and pathways such as glutathione peroxidase 4 (GPX4) can suppress ferroptosis by reducing lipid hydroperoxides in biological membranes [45]. Cancer cells seem to have adopted these pathways, and overexpression of GPX4 is a feature of many cancers, offering protection against oxidative stress-induced cell death [47]. Inhibition of GPX4 promotes ferroptosis in MDA-MB-231 and HD578T triple-negative breast cancer cells in vitro and increases sensitivity to EGFR inhibitors in vivo in xenograft tumors, suggesting that dual therapy with GPX4 inhibitors may enhance the anticancer effects of the treatment of lipid-rich tumors [48].

**Fig. 2:**
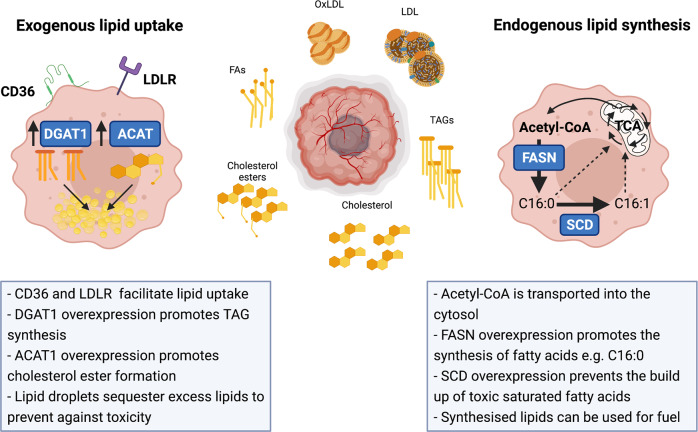
Tumor interstitial fluids (TIFs) contain a diverse array of lipids that promote tumor growth and metastasis. The tumor interstitial fluid is rich in a diverse array of lipids, including free fatty acids (FAs), low-density lipoproteins (LDLs), oxidized LDLs (OxLDLs), cholesterol, cholesterol esters and triacylglycerols (TAGs). Tumor cells express a range of cell surface receptors to facilitate the uptake of lipids, including the scavenger receptor CD36 and the LDL receptor. Overexpression of Diglyceride acetyltransferase 1 (DGAT1) is a common feature of many tumors. DGAT1 is responsible for the synthesis of TAGs from FAs, which can then be stored in lipid droplets (LDs). Acyl cholesterol acyltransferase (ACAT) is also overexpressed in many tumors. ACAT plays a key role in cholesterol homeostasis by catalyzing the formation of cholesterol esters from free cholesterol and FAs. Together, TAGs and cholesterol esters form the major components of LDs. LDs are a feature of many tumor cells and promote lipid homeostasis by sequestering excess intracellular lipids to prevent lipotoxicity, peroxidation and ferroptosis. Fatty acids may also be synthesised endogenously from cytosolic acetyl-CoA. Fatty acid synthase (FASN) expression is a feature of many cancers and catalyses the production of palmitic acid. Palmitic acid can be converted to the monounsaturated fatty acid (MUFA) palmitoleic acid to protect against the toxic effects of saturated fatty acids (SFAs) such as palmitic acid

While lipids are important structural components and essential fuel sources for many cancers, excess lipids may also be stored intracellularly in LDs. Indeed, LDs are a feature of many cancers, including glioblastoma [49] and prostate [50], colon [51], and renal [52] cancers. LDs are lipid-buffering organelles that sequester intracellular FAs in the form of TAGs to prevent lipotoxicity [7]. DGAT1 is responsible for the synthesis of TAGs (Fig. 2) and is highly expressed in glioblastoma patient tissues, which correlates with poor patient survival [49]. Inhibition of DGAT1 in multiple glioblastoma cell lines and patient-derived GBM30 cells in vitro caused dysregulated lipid metabolism, triggering the accumulation of acylcarnitines and the production of ROS, resulting in apoptosis. Indeed, genetic inhibition of DGAT1 in vivo suppresses tumor growth and prolongs the survival of glioblastoma-bearing mice, demonstrating that targeting DGAT1 may offer therapeutic benefit for patients with glioblastoma [49]. It was recently shown that LDs preferentially store n-3 and n-6 PUFAs in cancer cells, possibly due to their capacity to undergo peroxidation. However, excess PUFAs that surpass LD storage exert cytotoxic effects in cancer cells by inducing ferroptosis in vitro [53]. Interestingly, a fish oil-based diet containing a high proportion of the n-3 PUFA docosahexaenoic acid (DHA) induced ferroptosis and delayed tumor growth in vivo in a mouse xenograft model of colorectal cancer. Inhibiting DGAT further increased the levels of peroxidation in mice fed a DHA-rich diet [53], again highlighting a role  for specific dietary regimens when used in combination with traditional cancer therapies.

In addition to PUFAs, saturated fatty acids (SFAs) have also been implicated in driving cellular lipotoxicity through ER stress [54]. To prevent against the toxic effects of SFAs, cancer cells must maintain an optimal ratio of SFAs to monounsaturated fatty acids (MUFAs) intracellularly. Stearoyl-CoA desaturase (SCD) is an enzyme that catalyses the preferential conversion of palmitic acid (C16:0) and stearic acid (C18:0) to palmitoleic acid (C16:1) and oleic acid (C18:1), respectively. The SCD protein is highly expressed in many human cancers (Fig. 2), including human colon, esophageal and liver cancers [55]. SCD consumes excess SFAs, which maintains cell viability and survival by preventing SFA-induced ER stress [55]. As C16:0 is the main product of FASN, cancer cells commonly co-overexpress FASN and SCD to maintain homeostatic levels of SFAs and MUFAs [56]. In addition, SCD activity increases the ratio of MUFAs to PUFAs in cellular membranes. Thus, heightened SCD activity is advantageous to cancer cells, as it results in fewer peroxidation-susceptible targets [57].

While aberrant and dysregulated lipid metabolism is associated with ROS production, β-oxidation provides a valuable source of NADPH to maintain cellular redox balance. The oxidation of acetyl-CoA-derived citrate in the TCA cycle is a major source of NADPH, which serves to protect against ROS-induced toxicity and supports tumor cell survival during energy stress through AMPK signaling [58]. Therefore, cancer cells must maintain an optimal rate of β-oxidation whereby the beneficial effects of NADPH exceed the levels of ROS-induced cellular damage.

### Lipids and immunity

The metabolic challenges imposed by the TME mediate immune cell dysfunction and exhaustion, which drive tumor progression. The current dogma is that tumor cells comfortably outcompete immune cells for glucose [59] and glutamine [34], resulting in vast areas devoid of these nutrients [60], while lipids are readily available for use [3, 4]. However, a recent paper by the Rathmell laboratories surprisingly found that glucose is highly available in a range of cancer models [2]. In a sense, this is unsurprising, as FDG-PET imaging operates on the basis that tumor cells have heightened glucose uptake. However, in contrast to the current understanding, intratumoral myeloid cells have the greatest capacity to consume glucose, followed by T cells and tumor cells. The experiments were performed using state-of-the-art methods to trace the metabolism of glucose and other nutrients in vivo [2]. This study prompts us to question the notion that nutrient competition exists between tumor and immune cells due to restricted nutrient access. It is also possible that even when glucose is available to all cell types, heightened levels of aerobic glycolysis in tumor cells generate toxic biproducts, including lactate, which has been shown to influence immunity in many TMEs [61, 62]. Despite this new evidence, and although lipids were not the focus of this study, the authors reported that lipids were predominantly taken up by CD45^-^ tumor cells [2]. In addition, it is often found that intratumoral immune cells rewire their metabolism toward lipid uptake and oxidation in the TME, which is consequently linked to their dysfunction. Thus, a lipotoxic TME functions as a protumor environment, promoting tumor proliferation while impairing cytotoxicity and promoting immunosuppression.

### Lipids suppress antitumor immunity

#### CD8 T cells

In response to immune challenge, CD8 T cells increase glucose uptake and display heightened rates of glycolysis to support cytolytic effector functions [63]. High levels of exogenous glucose are imperative to sustain the transcription of glycolytic machinery and effector cytokines [64, 65]. In established tumors, which are often reported to have low levels of glucose and high levels of hypoxia, CD8 T cells become metabolically and functionally exhausted [59]. Limited access to glucose may force a metabolic switch toward FA uptake and metabolism in CD8 T cells to preserve energy homeostasis and ATP production. This metabolic adaptation is consequently linked to CD8 T cell dysfunction across many human cancer types and mouse models.

Pancreatic ductal adenocarcinoma (PDA) is an example of a lipid-rich tumor with a poor overall survival rate [66]. Using the KC mouse model of PDA, which closely recapitulates human PDA progression, Manzo et al. found that CD8 T cells acquire features of functional exhaustion: upregulation of PD-1 and TIM-3 and reduced production of IFN-γ and granzyme B. In response to the nutrient composition of the PDA TME, CD8 T cells increase the expression of the specialized lipid transporter CD36 and accumulate LCFAs. Despite this change, intrapancreatic CD8 T cells fail to engage in OXPHOS and downregulate very long chain acyl-CoA dehydrogenase (ACADVL), which catalyzes the first step of β-oxidation, leading to metabolic exhaustion. Increasing ACADVL expression improved the survival and metabolic fitness of CD8 T cells in this metabolically hostile TME in vivo, highlighting the possible importance of coupling lipid uptake with β-oxidation to promote CD8 T cell survival in the context of lipid-rich tumors [8].

CD36 expression and associated metabolic exhaustion are common features of intratumoral CD8 T cells across multiple cancer models and types. Indeed, intratumoral CD8 T cells from B16 models of melanoma also progressively increase the expression of CD36 [4, 67], possibly as a function adaptation to heightened lipid availability [3]. Like what happens to CD8 T cells in PDAs, lipid uptake through CD36 induces terminal exhaustion and is associated with PD-1 and TIM-3 expression and decreased production of IFN-γ and TNF-α in CD8 T cells from B16 melanomas. CD36 knockout (KO) mice maintain CD8 T cell polyfunctionality through the co-production of IFN-γ and TNF-α, which abrogates tumor growth [4, 67]. In addition to FA uptake, CD36 mediates the uptake of oxidized lipids, including OxLDL. OxLDL enhances lipid peroxidation in CD8 T cells, resulting in ferroptosis. Overexpression of GPX4 to prevent lipid peroxidation or direct inhibition of ferroptosis enhanced IFN-γ and TNF-α production by CD8 T cells in vivo [4]. While tumor cells have developed mechanisms to overcome lipotoxicity and peroxidation [47], these studies reveal that intratumoral CD8 T cells are sensitive to ferroptosis and that GPX4 may be a novel target for CD8 T cell-based therapies. Together, these studies highlight a detrimental effect of lipid uptake through CD36 on CD8 T cell function and stress the therapeutic potential of blocking CD36 to boost CD8 antitumor immunity.

In addition to fatty acids and oxidized lipids, the TME is enriched with cholesterols and cholesterol esters [4]. Intratumoral cholesterol ester accumulation is a feature of breast cancer and drives tumor cell proliferation and tumor aggression [14]. On the other hand, cholesterol induces immune checkpoint expression, ER stress and functional exhaustion of intratumoral CD8 T cells from B16 mouse models of melanoma. Inhibiting the ER stress sensor XBP1 in CD8 T cells was sufficient to overcome the effects of cholesterol and enhance IFN-γ, TNF-α and granzyme B production in vivo [68]. However, an important aspect to consider is the differences between intracellular and plasma membrane cholesterol. Plasma membrane cholesterol promotes CD8 T cell immunological synapse formation and maturation, TCR clustering and signaling, which together allow efficient and directed target cell killing in vitro. Preventing cholesterol ester formation by inhibiting ACAT1 in CD8 T cells increases plasma membrane cholesterol levels and in turn augments lytic granule and inflammatory cytokine production. Indeed, ACAT1 inhibition in B16 melanoma-bearing mice promoted CD8 T cell tumor infiltration while enhancing effector function and proliferation in vivo. Moreover, dual therapy with an ACAT1 inhibitor and anti-PD-1 agent was better than either therapy alone at inhibiting tumor progression in vivo [69]. Together, these data highlight the importance of cholesterol location in CD8 T cell function. Targeting ACAT1 to prevent cholesterol ester formation may impair tumor growth while promoting the production of cytokines and cytolytic granules by CD8 T cells.

Given the dual role of lipids in promoting tumor proliferation and immunosuppression, it is unsurprising that an HFD accelerates tumor growth and increases tumor burden [40, 70]. Indeed, a HFD promotes mammary tumor progression by inducing β-oxidation in intratumoral CD8 T cells in a STAT3-dependent manner. In this setting, adipocyte-secreted leptin activates STAT3 signaling in proximal CD8 T cells to suppress glycolysis and promote β-oxidation, resulting in impaired IFN-γ and granzyme B production. Genetic ablation of STAT3 slowed tumor development by enhancing CD8 T cell infiltration and effector function in vivo [70]. It is plausible that mammary adipocytes secrete lipids to promote lipid uptake and β-oxidation and suppress CD8 T cell effector function, as is the case with omental adipocytes and human ovarian cancer metastasis [17].

In contrast to these reports demonstrating the deleterious effects of lipids on CD8 T cell function and metabolism in the TME, FAs have also been implicated in supporting CD8 T cell function within a metabolically challenging TME. In a mouse model of B16 melanoma that has a high abundance of FAs, promoting β-oxidation in CD8 T cells was shown to improve T cell polyfunctionality and act in synergy with anti-PD-1 therapy to efficiently delay tumor growth [3]. The different metabolic statuses of the TMEs and different tumor types may explain these opposing findings on the role of β-oxidation in promoting or suppressing CD8 T cell antitumor responses. It is important to note that the TIF of B16 tumors contains a diverse array of lipids, including FAs, acylcarnitines, cholesterols and triacylglycerols [4]. It is possible that these contrasting reports are due to the different cocktails of lipids in the TME, which may differentially affect CD8 T cells. As we will discuss in a later section, distinct FA species differentially affect tumorigenesis and may also differentially regulate antitumor immunity. Finding the ideal composition of lipids in the TME to harness an effective antitumor response would prove beneficial for the treatment of many cancers in which lipids are central to tumor and T cell metabolism.

#### NK cells

Natural killer (NK) cells are effector lymphocytes that rapidly coordinate innate and adaptive antitumor immune responses and are the first line of defense against malignant cells. NK cells are potent producers of IFN-γ and directly kill tumor cells through the targeted release of cytotoxic perforins and granzymes [71]. Glucose metabolism is important for the control of the responses of NK cells, which use an unconventional metabolic configuration involving the citrate-malate shuttle (CMS) to fuel OXPHOS [72]. NK cell proliferation and effector functions are controlled by SREBP transcription factors that sustain activity through the CMS [73]. Conventionally, SREBPs are master regulators of de novo lipid synthesis [74]. However, SREBP-dependent metabolic reprogramming of NK cells does not involve their traditional role in controlling lipid synthesis [73]. Rather, SREBP maintains glucose metabolism in NK cells by controlling the transcription of Slc25A1 and ACLY, two key factors required for CMS activity. SREBPs are inhibited by cholesterol and oxidized forms of cholesterol (oxysterols, including 25-hydroxycholesterol (25-HC) and 27-HC) [75]. Indeed, treatment of NK cells with 25-HC prevents NK cell metabolic reprogramming, which impairs their ability to kill K562 tumor cells and produce IFN-γ in vitro [73]. In addition to cholesterol and cholesterol esters, oxysterols are a common signature of TMEs and can be secreted by cancer cells themselves, likely to impair antitumor immune responses [76, 77]. 27-HC is elevated in ER^+^ breast cancer tissue, which promotes tumor cell proliferation and facilitates breast cancer metastasis [15, 78]. Peripheral NK cells from patients with metastatic breast cancer are functionally defective and metabolically impaired, resulting in reduced killing capacity [79]. It is possible that 27-HC contributes to NK cell metabolic dysfunction in patients with metastatic breast cancer by targeting Srebp transcription factors; however, this notion has not yet been considered in detail.

The anabolic protein complex mTORC1 regulates NK cell metabolic reprogramming and is essential for the production of IFN-γ and granzyme B [80]. Our laboratory has shown that the FAs palmitate and oleate robustly decrease mTORC1 activity in NK cells while activating a PPAR-dependent lipid metabolism program. This transcriptional switch induces a state of metabolic paralysis, impairs IFN-γ production, and reduces NK cell antitumor activity in vivo in the B16 mouse model of melanoma. However, blocking fatty acid transport into the mitochondria with etomoxir is sufficient to at least partially rescue NK cell cytotoxicity [40]. In line with these findings, NK cells derived from lymphoma-bearing mice and patients with diffuse large B cell lymphomas (DLBCL) display increased lipid metabolism, impaired mTORC1 activation and reduced production of IFN-γ. The lymphoma environment is rich in lipids, which supports a shift toward lipid metabolism and disrupts an effective antitumor response [81]. These results further demonstrate how a metabolic adaptation to a lipid-rich environment can be consequently linked to cellular dysfunction and that metabolically rewiring NK cells away from β-oxidation may improve cancer outcomes in the context of a lipotoxic environment.

### Lipids support immunosuppression

While the evidence thus far describes a protumorigenic role for lipids by which they support tumor proliferation and inhibit cytotoxic lymphocyte activities, not all immune cells are affected by lipids in the same way. Indeed, recent evidence suggests that excess lipids in the TME support suppressive or regulatory immune cells that could further promote a protumor environment.

#### Tregs

Regulatory T cells (Tregs) are a major immunosuppressive population that represent a significant obstacle for antitumor immunity. High infiltration of Tregs is associated with poor clinical outcome in many mouse and human cancers, including colorectal and ovarian cancers [82–84]. Like other cells in the TME, Tregs adjust their metabolic configuration to support their survival and function in such a hostile environment. Compared to peripheral blood mononuclear cells, intratumoral Tregs from patients with melanoma, breast cancer, and non-small-cell lung carcinoma express high amounts of the fatty acid scavenger receptor CD36. CD36 orchestrates Treg metabolic reprogramming and mitochondrial fitness in the TME by providing lipids to induce PPAR-dependent lipid metabolism, which promotes Treg viability and suppressive functions. Indeed, Treg-specific genetic ablation of CD36 attenuated tumor growth in a mouse model of melanoma and promoted Treg apoptosis while reinforcing CD8 T cell infiltration and effector cytokine production. Importantly, CD36 KO did not promote peripheral autoimmunity, demonstrating that this metabolic configuration involving lipid uptake and metabolism is unique to intratumoral Tregs, highlighting the potential for targeting lipid metabolism for cancer therapy [61].

In addition to lipid uptake, it was recently reported that intratumoral Tregs synthesize fatty acids and cholesterol de novo by upregulating a SREBP transcriptional signature. SREBPs are essential to maintain Treg viability and immunosuppression in the TME, as SCAP deletion to inhibit the expression of SREBP target genes increased the frequency of IFN-γ^+^ Tregs in the tumor while also increasing the number of TNF-α- and IFN-γ^+^ polyfunctional CD8 T cells. Together, these effects significantly reduced tumor growth in mice with MC38 colon adenocarcinoma and B16 melanoma, highlighting the importance of lipid synthesis and metabolism in maintaining intratumoral Treg immunosuppression in multiple cancer models [85]. Importantly, both of these papers indicated a dispensable role for lipid metabolism in Treg-mediated immune homeostasis in the periphery and identified a context-specific lipid signature for Tregs in the TME. Therefore, targeting lipid metabolism in Tregs would unleash an effective antitumor immune response without affecting peripheral Tregs.

#### γδ T cells

γδ T cells are a small population of poised innate lymphocytes capable of producing the key effector cytokines IFNγ and IL-17. In mice, γδ^IFNγ^ cells express CD27 and mediate an effective antitumor response through the production of IFN-γ and direct tumor cell killing via perforin and granzymes. In contrast, many studies have described a protective role for γδ^17^ cells in cancer, and γδ^17^ cells recruit other immunosuppressive immune cells, including neutrophils, to enhance tumor growth [86] and metastasis [87]. The rigid IFNγ-IL-17 dichotomy between γδ T cell effector subsets is established in the thymus during γδ T cell development, and we have recently shown that this is driven by distinct metabolic configurations. Like other effector cells, such as CD8 T cells and NK cells, γδ^IFNγ^ cells almost exclusively use glycolysis. However, γδ^17^ cells engage in oxidative lipid metabolism, which is supported by a higher mitochondrial mass and potential [88]. We and others recently demonstrated that γδ^17^, but not γδ^IFNγ^ cells, accumulate when mice are fed high-fat (HF) diets [88] and ketogenic diets (KDs) [89], suggesting that a lipid-rich environment supports γδ^17^ cell expansion. Indeed, immunosuppressive γδ^17^ cells are enriched in B16 melanoma tumors, which harbor a diverse array of lipids [3, 4]. Mice fed a HFD had increased γδ^17^ cell tumor infiltration compared to mice fed a standard-fat diet, and this increased infiltration correlated with enhanced tumor growth. Together, these data highlight the role of lipid uptake and metabolism in γδ^17^ cells and show that lipid-rich environments support the accumulation of immunosuppressive γδ^17^ cells, which promote tumor growth.

Hypercholesterolemia is a risk factor for the development, metastasis, and recurrence of ER^+^ breast cancers and is driven in part by the oxysterol 27-HC [15, 90]. In addition to its protumorigenic properties, 27-HC has recently gained traction as an immunomodulatory metabolite. 27-HC increases the number of γδ T cells and neutrophils in primary E0771 breast tumors and lung metastases while reducing the number of cytotoxic CD8 T cells at both sites [90]. Neutrophils are essential for breast cancer metastasis [91] but require γδ T cells for induction of their full protumorigenic properties, as γδ T cell ablation attenuates 27-HC-induced metastasis and colonization [90]. It is likely that 27-HC promotes the expansion of γδ^17^ cells, and not γδ^IFNγ^ cells, as IL-17 production from γδ T cells induces neutrophil polarization and expansion to promote breast cancer metastasis [87]. As γδ^17^ cells primarily engage in oxidative lipid metabolism, it would be interesting to investigate whether 27-HC induces lipid uptake in γδ T cells to fuel IL-17 production and promote metastasis.

#### Tumor-associated macrophages

Macrophages are specialized phagocytic cells that exist along a heterogenic spectrum of functional and metabolic phenotypes. HIF-1α coordinates a glycolytic program in inflammatory antitumorigenic macrophages, resulting in the production of reactive oxygen species (ROS) and effector cytokines, including IL-1β [92], which mediate tumor killing and inhibit angiogenesis. Anti-inflammatory macrophages, on the other hand, are regulated by PPARγ [93] and STAT6 [94], engage in oxidative lipid metabolism, and highly express arginase-1, IL-4 and IL-10, which together resolve inflammation and promote tissue repair [95]. Intratumoral macrophages (coined tumor-associated macrophages or TAMs) are phenotypically similar to anti-inflammatory macrophages and constitute the main population of CD45^+^ cells in solid tumors [96]. A high proportion of TAMs is a poor prognostic marker for many cancers, including melanoma [97] and pancreatic [98] cancers, especially when coupled with reduced numbers of CD8 T cells, due to the lack of cytotoxicity and ability to promote tumor invasion through the production of VEGF to induce angiogenesis and shape the TME [99].

It is well established that tumor-derived factors, such as lactic acid, play a role in polarizing naïve macrophages into TAMs to encourage tumor growth and dissemination [100]. Recently, it has also emerged that lipids are crucial for the differentiation and generation of immunosuppressive, tumor-promoting TAMs across multiple human and mouse cancers. This finding is not particularly surprising, as anti-inflammatory macrophages engage in lipid metabolism programs to support their functions. Indeed, TAMs from human melanoma and colon cancer tissues as well as from mice bearing 5TGM1, EL4 or B16 melanoma tumors highly express CD36, which leads them to have enhanced lipid uptake compared to naïve macrophages. Interestingly, treating in vitro-generated TAMs with etomoxir to inhibit β-oxidation significantly reduces the expression of TAM-associated genes (including *Arg1*, *Vegf*, and *Pparg*), while TAM-specific CD36 KO mice successfully contained EL4 tumor growth, demonstrating that targeting lipid metabolism in TAMs may be an effective strategy to combat tumor growth in vivo [101]. Interestingly, it was shown that the long-chain unsaturated fatty acid oleic acid polarizes naïve macrophages into immunosuppressive TAMs in vitro by augmenting mitochondrial respiration. Importantly, these effects were not recapitulated by the long chain saturated fatty acid stearic acid, revealing a specifically immunosuppressive role of oleic acid [102]. TIF of B16 melanoma tumors contains an array of long chain unsaturated fatty acids [3]. Determination of whether these other long-chain fatty acids also play a role in TAM polarization in the TME may reveal how specific lipids regulate immunosuppression in the TME.

### Dietary modulation in cancer

It is becoming increasingly evident that the nutritional state of a patient plays a role in their response to cancer therapy. Preclinical evidence suggests that dietary restriction of particular nutrients, including glucose, fructose, methionine and serine, may enhance cancer therapy [103]. Given that lipids are emerging as key nutrients fueling cancer proliferation, it is not surprising that modulation of dietary intake of fat is also gaining much interest in cancer studies. We and others have shown that HFD-induced obesity accelerates the growth of multiple tumor models, including the B16 melanoma and MC38 colorectal adenocarcinoma models [40, 104]. However, little is known about the role of individual dietary lipid components or how the source of dietary fat regulates tumor growth and antitumor immunity, but research on the role of common dietary SFAs and MUFAs in tumorigenesis is accumulating.

### Obesity and the role of dietary fat

It is estimated that 2 billion adults are overweight or obese worldwide [105]. Obesity is an established risk factor for the development of at least 14 types of cancers, including esophageal, breast and liver cancers, and up to 50% of endometrial cancers [106, 107]. As previously mentioned, hypercholesterolemia is intimately linked with obesity and is an independent risk factor for breast cancer incidence, metastasis, and recurrence [13]. In addition to its association with increased circulating cholesterol, obesity is associated with increased plasma nonesterified fatty acids (NEFAs), which strongly predict cancer mortality [108]. NEFAs can freely diffuse into the liver, where they directly contribute to the pathogenesis of nonalcoholic fatty liver disease (NAFLD) [109]. NAFLD is a common comorbidity of obesity and is strongly associated with the development of hepatocellular carcinoma (HCC) [110]. This link between obesity, circulating lipids and cancer is supported by findings showing that weight loss following bariatric surgery decreases circulating cholesterol [111] and NEFAs [112] while also reducing cancer risk [113].

While the causes of obesity are multifactorial, the overconsumption of dietary fat is a risk factor for cancer development in obesity. One of the first reports demonstrating the effect of dietary fat on cancer incidence was published in 1930. Watson and Mellanby reported that adding butter to rodent diets accelerated tar-induced skin cancer growth [114]. This finding has since been consistently supported by experiments using rodent models of HFD-induced obesity, leading to the conclusion that overconsumption of dietary fat is positively associated with cancer risk [115]. As distinct FFA species differentially affect signaling pathways involved in cell proliferation [115], it is likely that the fatty acid composition of dietary fats may play a role in tumor growth and progression in obesity. Here, we discuss 3 key FFAs as an example, although there are many more lipid species of interest, each of which may have differential effects on cancer and immunity.

Palmitic acid (C16:0) is the most abundant SFA in the human body and can be obtained through diet intake or synthesized endogenously. Palmitic acid is enriched in many common fat sources, including palm oil, butter, lard, and olive oil [116]. There is increasing evidence that palmitic acid participates in the pathogenesis of many cancers through palmitoylation of oncoproteins and promotion of cell proliferation. Palmitic acid provides a substrate for palmitoylation reactions, a post-translational protein modification that alters the location, structure, and function of many proteins [30]. NRAS is a proto-oncogene that regulates cell growth and proliferation and has been implicated in leukemogenesis. Palmitoylation of NRAS promotes its localization to the plasma membrane and subsequent hyperactivation of downstream signaling pathways, such as the Akt and Erk1/2 pathways, to support cell growth. Palmitoylation is essential for oncogenic NRAS signaling, as mice transplanted with cells overexpressing a nonpalmitoylatable NRAS mutant remain healthy and do not develop leukemia [117]. Many studies have demonstrated that the protumorigenic effects of a HFD are due to an increased abundance of palmitic acid. Like HFD feeding, administration of palmitic acid itself promoted the proliferation of colorectal cancer (CRC) cells in vitro by enhancing the expression of β2-adrenergic receptor, which is essential for promoting CRC growth [118]. In addition, exposing intestinal organoids to palmitic acid in vitro recapitulated the effects of HFD feeding by increasing the number of Lgr5^+^ intestinal stem cells, which boosted their ability to spontaneously form colorectal adenocarcinomas [41]. As palmitic acid is abundant in many common sources of fat, restriction of dietary palmitic acid would be a challenge. Therefore, identifying the molecular targets and signaling pathways that are regulated by palmitic acid may help design effective targeted therapies.

Stearic acid (C18:0) is another example of a long-chain SFA enriched in butter, lard, and cocoa butter [116]. The effects of stearic acid on tumorigenesis are less studied than those of palmitic acid; however, evidence points toward a protective role of stearic acid against some cancers. Epidemiological evidence suggests that dietary intake of stearic acid is inversely associated with the risk of breast cancer development in postmenopausal women [119]. These findings are supported by in vitro data showing that stearic acid inhibits the proliferation of human breast cancer cells [120] by increasing the expression of key cell cycle inhibitors p21 and p27 [121]. Furthermore, dietary stearic acid reduced the tumor burden in a breast cancer carcinogen model by inhibiting key cell cycle checkpoints in vivo [121]. Stearic acid preferentially induced apoptosis in human breast cancer cells over noncancerous breast cells, revealing a specific antitumorigenic role of stearic acid [122]. Despite these antitumorigenic properties of stearic acid, the effects of stearic acid on the immune system are not known. We and others have shown that LCFAs, including palmitic acid, impair NK cell [40] and CD8 T cell [8] function and metabolism in vitro. Given that stearic acid is another (longer) LCFA, it is plausible that stearic acid may also impair antitumor immunity; however, this hypothesis has not been examined in detail. Increasing stearic acid consumption to inhibit tumor burden may pose challenges, as fat sources of stearic acid also contain high amounts of palmitic acid, which stimulate tumor growth. It was recently reported that the stearic acid-based ester conjugate propofol stearate inhibited the growth, migration, and adhesion of human breast cancer cells in vitro [123]. These lipophilic drugs may be used in combination with other chemotherapies, offering an effective strategy to reap the anticancer benefits of stearic acid without the drawbacks of palmitic acid, although their effects on cytotoxic lymphocytes need to be considered.

Oleic acid (C18:1) is the most abundant MUFA in the human diet. Oleic acid accounts for ~20% of FAs in all fat sources and is highly enriched in olive oil, accounting for almost 80% of the FAs in olive oil [116]. Olive oil is a main component of the Mediterranean diet [124], which is renowned for its health benefits and is inversely associated with liver, breast and colorectal cancer mortality [125]. Olive oil consumption itself has beneficial effects in counteracting liver steatosis [126] and is associated with a decreased risk of having all types of cancer [127]. It is thought that the beneficial effects of olive oil are due to its high proportion of oleic acid, which has potent antitumor effects. For example, oleic acid reduces the viability, migration and invasion of Hep3b and Huh7.5 cells in vitro without affecting healthy hepatocytes, highlighting a specific antitumorigenic role of oleic acid [128]. Furthermore, intraperitoneal injection of oleic acid reduced the growth of CAL27 squamous cell carcinoma xenograft tumors in vivo. Whether this protective role of oleic acid is sustained when it is administered orally was not examined [129]. While these data illustrate an antitumorigenic role of oleic acid, it is important to note that the benefits of a Mediterranean diet can also be attributed to many bioactive components with cancer-preventative properties, including polyphenols and triterpenes [130].

### Obesity, fatty acids, and immunity

NK cells and CD8 T cells from obese mice and humans accumulate lipids and activate a lipid metabolism transcriptional program. This metabolic adaptation is linked to their dysfunction, resulting in impaired cytotoxicity, cytokine production and metabolic paralysis [40, 81]. Culturing NK cells with palmitic acid in vitro recapitulates the metabolic and functional defects caused by obesity, demonstrating an added protumorigenic effect of palmitic acid [40]. Interestingly, treating NK cells in vitro with a combination of palmitic and oleic acid further impaired their function and metabolism [40], demonstrating that the anticancer effects of oleic acid may not translate into a beneficial effect on immunity. While little is known about the differential effects of individual FAs on NK and CD8 cell functional and metabolic responses, specific monounsaturated and polyunsaturated FAs that accumulate in atherosclerotic plaques of *Apoe-/-* mice have been shown to selectively stimulate macrophage foam cell formation. Oleic, linoleic, and arachidonic acids augment *IL-1α* but not *IL-1β* production by macrophages in vitro and in vivo (only shown for oleic acid), which contributes to vascular inflammation [131]. Despite these proinflammatory effects of oleic acid on macrophages in atherosclerotic plaques, oleic acid has been shown to induce an anti-inflammatory TAM phenotype in vitro, characterized by the expression of *Vegf*, *Arg1* and *Mmp9* in addition to *IL-1α*. These context-dependent roles of oleic acid in macrophage function may be explained by factors released by tumor cells into the TME, such as lactic acid, which has been extensively shown to encourage TAM generation and immunosuppression [100].

Given these protumorigenic effects of lipids on TAMs in the TME, it is unsurprising that obesity and dietary intake of fat affects macrophage polarization states in the periphery. Indeed, transcriptome profiling of resident macrophages from human, tumor-free breast tissue of obese individuals indicates that these cells adopt a gene signature resembling that of anti-inflammatory TAMs. Interestingly, in vitro experiments show that the remodeling of the breast extracellular matrix that occurs in obesity, possibly due to adipose fibrosis, controls the functional state of resident macrophages [132]. It is possible that a spillover of lipids (particularly oleic acid) from fibrotic adipocytes in breast tissue is responsible for these changes in the macrophage phenotype that occur in obesity and may explain the link between obesity and breast cancer.

**Fig. 3:**
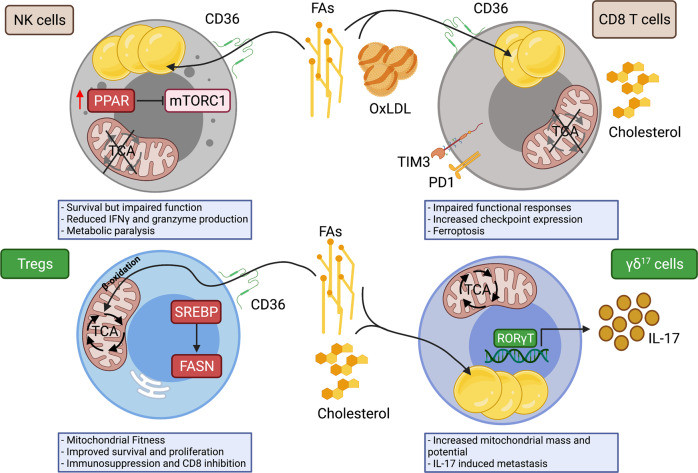
Lipids differentially affect immune populations while overall promoting an immunosuppressive phenotype. Lipids have emerged as an enemy of an antitumor environment because they suppress cytotoxic antitumor NK and CD8 T cells while promoting the survival and protumorigenic roles of Tregs and γδ^17^ cells. In the presence of FAs, NK cells display increased expression of the scavenger receptor CD36, which leads to the accumulation of intracellular lipids. In response to lipids, NK cells activate a PPAR-dependent lipid metabolism transcriptional program, which leads to the inhibition of the mTORC1 protein complex. Loss of mTORC1 signaling results in a state of metabolic paralysis, resulting in reduced cytotoxicity and impaired IFNγ and granzyme B production. CD8 T cells also display increased expression of CD36 and markers of functional exhaustion, including PD-1 and TIM-3, in lipid-rich tumor environments. In addition to FAs, CD8 T cells may also transport OxLDL through CD36, which enhances lipid peroxidation, resulting in ferroptosis. Together, these metabolic adaptations to a lipid-rich environment result in impaired functional responses and cultivate a protumorigenic environment. On the other hand, lipid uptake and synthesis have been shown to promote Treg immunosuppression in many tumor models. Intratumoral Tregs display heightened expression of CD36, which provides lipids to activate a PPAR-dependent lipid metabolism program that is essential for their survival, proliferation and protumor functions. Intratumoral Tregs also increase the expression of SREBPs, leading to the de novo synthesis of FAs and cholesterol. Similarly, protumorigenic γδ^17^ cells rely on lipid uptake and metabolism to regulate IL-17 production. γδ^17^ cells display increased mitochondrial mass and potential to support heightened rates of lipid metabolism. IL-17 is important for promoting neutrophil-mediated cancer metastasis, highlighting the role of lipids in promoting primary and metastatic cancer progression

The TME is rich in a diverse array of fatty acids, acylcarnitines, cholesterol esters and structural lipids, which increase in abundance with tumor progression [3]. It would be interesting to investigate how specific lipid cocktails regulate NK and CD8 T cells and immunosuppressive cells and whether this regulation corresponds to functional responses and metabolic configurations as the tumor progresses. In addition to FAs and cholesterols, acylcarnitines have emerged as potential regulators of metabolism in obesity. Incomplete β-oxidation of FAs is a feature of HFD-induced obesity that leads to the accumulation of medium- and long-chain acylcarnitines in the plasma [133]. While there are no reports on the effects of acylcarnitines on NK or CD8 T cells, acylcarnitines activate proinflammatory signaling pathways in macrophages in an acyl chain length- and concentration-dependent manner, resulting in the production of TNF-α and other macrophage inflammatory cytokines, such as IL-1β, MIP2 and MCP-1 [134]. Whether NK or CD8 T cell responses are affected by acylcarnitines has yet to be examined, but such studies may uncover an added layer of immunosuppression in the TME in obesity. Fig. 3.

## Concluding remarks and future directions

Tumor growth is regulated by nutrients in the environment. Here, we have highlighted the mainly protumorigenic role of lipids and proposed that diet composition may play a role in tumorigenesis in two ways: directly promoting tumorigenesis and fueling a protumor immune response over an antitumor immune response. Recent evidence suggests that alongside conventional cancer therapies, strategies that modify the host’s diet to target the metabolic vulnerabilities of tumors may have beneficial effects in inhibiting cancer progression [103]. Indeed, caloric restriction (CR) has been proven to inhibit tumor growth in a mouse model of PDAC by lowering the levels of lipids in the plasma and TIF. The KD is another example of a dietary regime that restricts carbohydrates. However, the KD had no effect on PDAC tumor growth, likely due to the high level of lipids supplied by this diet  [135]. Withdrawal of nutritional FAs to starve lipid-addicted tumors such as melanomas and PDACs may have therapeutic potential; however, to date, there is no evidence to suggest that dietary restriction of FAs protects against cancer formation.

As described, preclinical studies targeting lipid uptake or metabolism have revealed a promising avenue for cancer therapies. Currently, there are some modulators of lipid metabolism in phase 1 and 2 clinical trials for the treatment of cancer. TVB-260 is an orally administered, nontoxic, selective inhibitor of FASN. TVB-2640 is currently being explored in phase 1 clinical trials for the treatment of colon cancers and has recently entered phase 2 clinical trials for the treatment of Her2^+^ breast cancers [6]. In addition, TVB-2640 is in phase 2 clinical trials (FASCINATE-1 study) for the treatment of nonalcoholic steatohepatitis, which increases the risk of developing liver cancers, highlighting the relationship between lipids, metabolic perturbation, and cancer. Owing to the abundance of preclinical evidence, it is likely that new generations of drugs that target many aspects of lipid uptake and metabolism will be investigated as potential therapies to prevent and treat many cancers in the future.
